# Rhythms of risk: the intersection of clocks, cancer, and chronotherapy

**DOI:** 10.1172/JCI198780

**Published:** 2026-02-02

**Authors:** Rebecca M. Mello, Selma Masri, Katja A. Lamia

**Affiliations:** 1 Department of Molecular and Cellular Biology, Scripps Research Institute, La Jolla, California, USA.; 2 Department of Biological Chemistry, Chao Family Comprehensive Cancer Center, University of California, Irvine, Irvine, California, USA.

## Abstract

Circadian clocks govern daily rhythms in cellular and physiological processes, including cell cycle, DNA repair, metabolism, and immune function, that influence cancer development and treatment response. Disruption of circadian regulators either promotes or suppresses malignancy depending on tumor type and biological context. This duality likely reflects systemic rewiring of circadian physiology and direct interactions between clock components and oncogenic pathways. These insights hold clinical relevance for the field of chronotherapy, which seeks to enhance therapeutic efficacy and minimize toxicity by aligning drug administration with circadian rhythms or by targeting elements of the molecular clock. In this Review, we highlight the promise of integrating circadian biology into precision oncology and underscore the importance of cancer type–specific investigations to harness the full therapeutic potential of chronotherapy in cancer.

## Introduction

The study of circadian rhythms began with observations of plant movements and now spans a range of organisms — from cyanobacteria to humans — highlighting the universal role of biological clocks. Circadian regulation of essential processes like hormone secretion, sleep, metabolism, and immune response (1) is fundamental to human health. Circadian rhythms are synchronized by environmental cues, including light and food intake. In mammals, the central circadian pacemaker is the suprachiasmatic nucleus in the anterior hypothalamus. It receives light input from the retina (2), transmits circadian signals within the hypothalamus via synaptic and paracrine communication, and communicates circadian information to peripheral tissues indirectly via hormones, body temperature, and metabolites.

**Figure 1:**
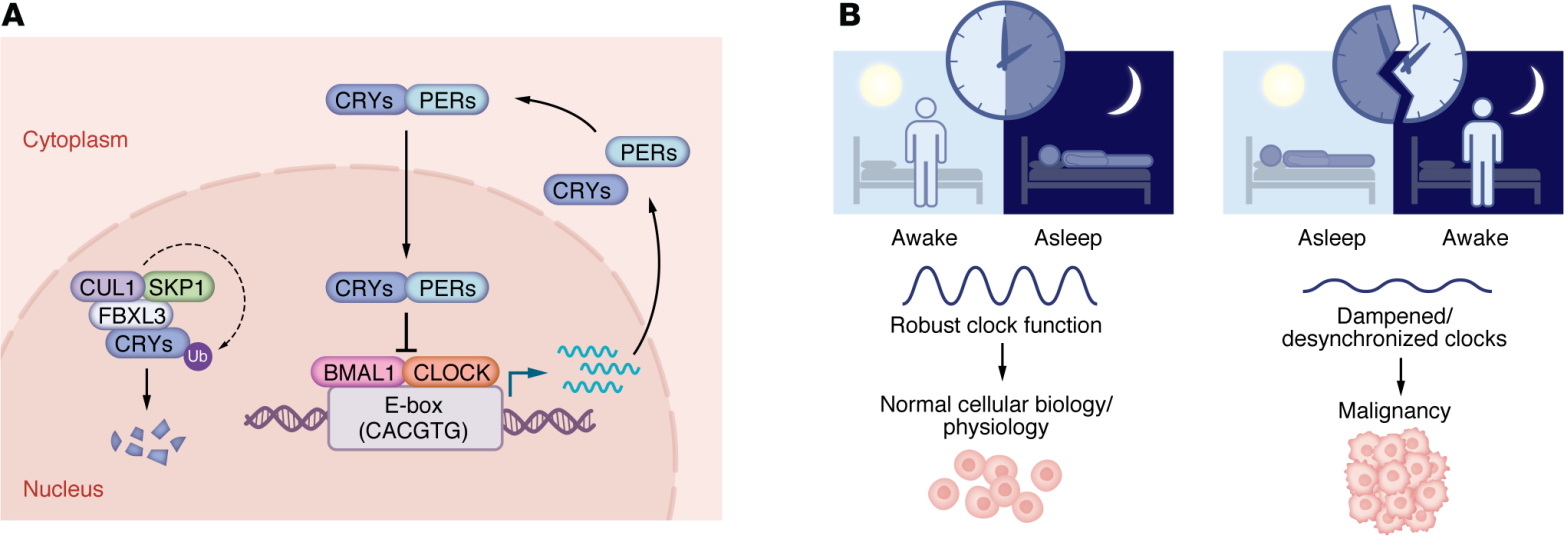
The molecular clock regulates physiological rhythms and influences cancer risk. (**A**) Core transcription–translation feedback loop driving daily oscillations in gene expression. BMAL1-CLOCK activates expression of clock-controlled genes (CCGs) through binding E-box sequences in chromatin. CCGs include CRYs and PERs, which repress BMAL1-CLOCK activity. CRYs are recruited to the SCF-FBXL3 complex, resulting in turnover required for timely reactivation of BMAL1-CLOCK activity. (**B**) Misalignment with environmental cues (right) increases susceptibility to several types of malignancy.

Cellular circadian rhythms involve a transcription-translation feedback loop in which the positive arm is driven by the heterodimer of basic helix-loop-helix PER-ARNT-SIM (bHLH-PAS) (3, 4) transcription factors BMAL1-CLOCK (5), which bind E-box sequences (5′-CACGTG-3′) to initiate the transcription of clock-controlled genes, including *PER1/2/3* and *CRY1/2*, which form the negative arm of the circadian clock by repressing BMAL1-CLOCK (6). Timely degradation of CRY, mediated by an F-box and leucine-rich repeat protein 3–containing (FBXL3-containing) Skp–Cullin–F-box (SCF) E3 ubiquitin ligase (7), resets the cycle (Figure 1A). Additional paralogs related to BMAL1 (i.e., BMAL2) and CLOCK (i.e., NPAS2) contribute to circadian rhythms in a tissue-selective manner and are less well understood (8–10). Nuclear hormone receptors in the 1D and 1F subfamilies (also known as REV-ERBs and RORs) regulate transcription of the gene encoding BMAL1, supporting circadian rhythms (11–13).

Epidemiological studies link circadian disruption to increased cancer risk (14, 15) (Figure 1B), leading the International Agency for Research on Cancer to classify night shift work as a probable human carcinogen (16). These studies underscore the link between circadian disruption and cancer but provide limited mechanistic insight, highlighting the need for further research to clarify the biological processes contributing to this phenomenon. Terms like “chronic jet lag,” “circadian misalignment,” and “clock disruption” are often used interchangeably, though they describe distinct perturbations: environmentally induced desynchrony, misalignment between internal and external cues, or molecular disruption of the clock, respectively. Such heterogeneity in terminology can obscure meaning and complicate interpretation across studies.

This Review explores recent insights into the circadian clock–cancer axis, focusing on how the biological pacemaker influences tumor biology and therapeutic responses in tissue-specific contexts.

## Multifaceted effects of circadian disruption on tumor growth

Environmental circadian misalignment (e.g., altered light–dark cycles) exacerbates tumor growth in murine models, including xenograft tumors grown from osteosarcoma or melanoma cells, spontaneous hepatocellular carcinoma, and genetically engineered colorectal, breast, and lung cancers (17–26); it does not affect tumor burden in a lymphoma model driven by transgenic expression of c-MYC (27). Mutation or deletion of circadian clock components can have oncogenic or tumor-suppressive consequences depending on the type of perturbation used, the cancer type, and the experimental model. Such variability may arise from tissue-specific, noncanonical functions of clock proteins, including CRY regulation of metabolic processes (28–30) and genome integrity (31–33) or BMAL1 heterodimerization with HIF2α (34, 35).

*BMAL1* deletion in human pancreatic cancer cells increases xenograft tumor growth in mice (36). Evidence in human and mouse cells suggests this may involve impaired apoptosis and enhanced cell cycle progression (36, 37). In genetically engineered mouse models of colon cancer, BMAL1 supports epithelial renewal and immune regulation; its loss accelerates tumor initiation (25), promotes tumor growth (24), and alters the tumor immune landscape (38). Similarly, BMAL1 loss in murine lung adenocarcinoma results in elevated c-MYC, increased proliferation, and metabolic dysregulation (21). Together, these findings suggest a tumor-suppressive role for BMAL1 in pancreatic, lung, and colon cancers.

Loss of the positive arm of the circadian clock suppresses tumorigenesis in other tumor types. In glioblastoma (GBM) cells derived from clinical samples, deletion of BMAL1 or CLOCK induces cell cycle arrest and apoptosis (39). Likewise, depletion of either *Bmal1* or *Clock* promotes stem cell differentiation and impairs tumor growth in murine acute myeloid leukemia (40). Loss of BMAL1 also reduces growth of clear cell renal cell carcinoma (ccRCC) human cells and xenograft tumors grown in mice. In ccRCC cells, BMAL1 promotes growth by dimerizing with HIF2α, thereby enhancing oncogenic signaling (34). BMAL1 overexpression increased migration of human breast cancer cell lines through upregulation of MMP9 (41), but the role of BMAL1 has not been investigated in an in vivo model for breast cancer. Knockdown of *BMAL1* was reported to reduce migration, invasion, and chemoresistance in colorectal cancer cells and clinical samples (42), in contrast to the studies demonstrating enhanced tumorigenesis upon deletion of *Bmal1* in *Apc*-deficient mouse models of colon cancer (24, 25, 38). Additional research is needed to determine whether *APC* dosage or other factors influence the impact of BMAL1 on colorectal cancer cell growth. *BMAL1* expression is lower in human colon adenocarcinoma than in normal colon and is higher in ccRCC samples than in normal kidney (34). Together, these findings suggest that BMAL1 and CLOCK can act as context-specific malignancy drivers.

While the mechanisms underlying this duality remain incompletely understood, they likely involve both malignant hijacking of circadian-regulated physiological processes and direct interactions between circadian clock components and cancer effectors. Importantly, the effects of circadian disruption may be shaped by host genetics, such that the same clock factor could exert opposing roles in different settings. Recent reviews caution against universal claims regarding circadian genes in cancer, emphasizing locus-, tissue-, and model-specific effects (43).

## Circadian regulation of pathways critical for tumor progression

**Figure 2:**
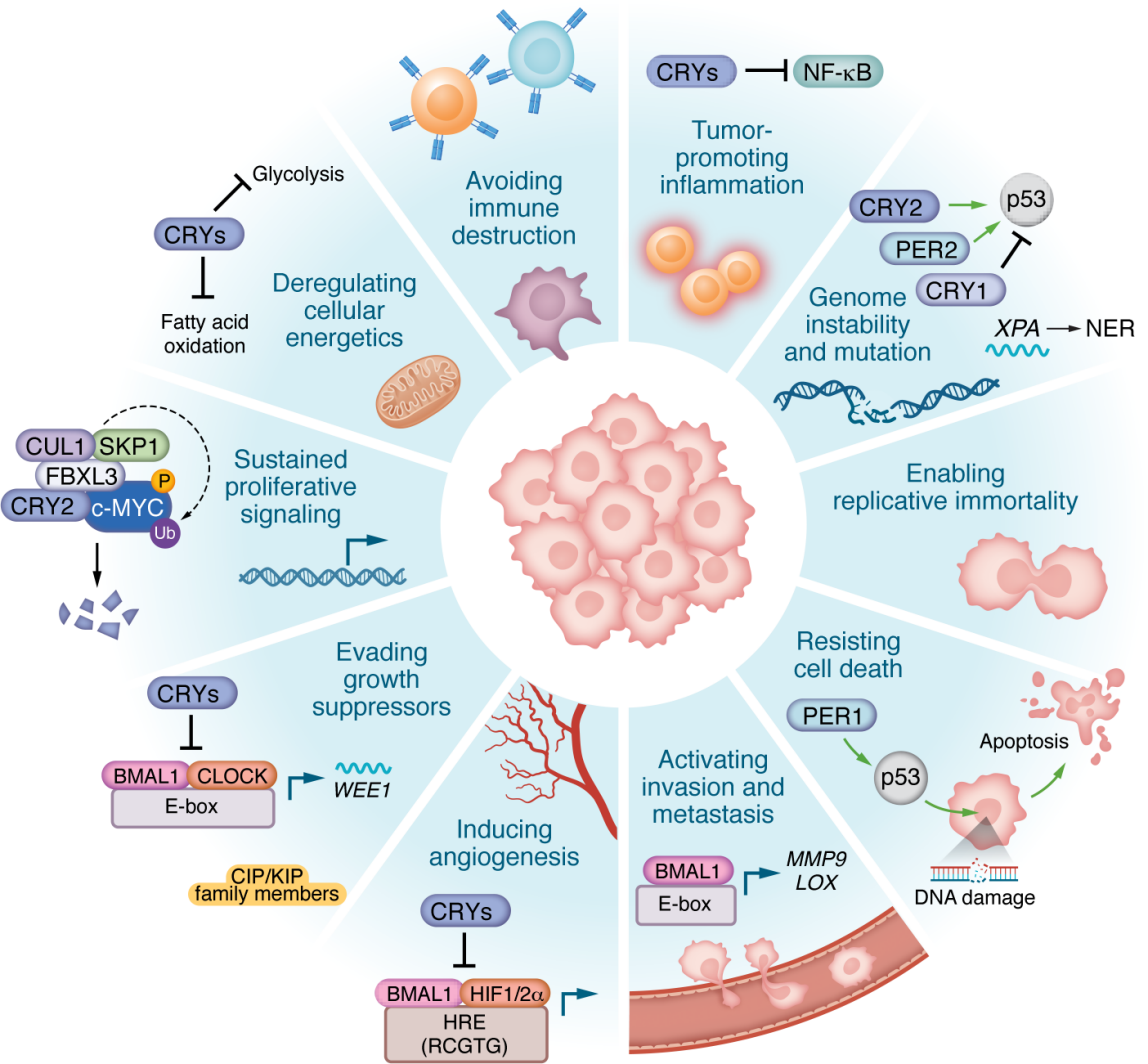
Circadian regulation of cancer hallmarks. The circadian clock interfaces with hallmarks of cancer, influencing proliferation, survival, genomic stability, metabolism, immune evasion, angiogenesis, invasion, metastasis, and tumor-promoting inflammation. These interactions highlight the broad impact of circadian rhythms on tumor initiation, progression, and therapeutic response. For example, CRY2 can facilitate the turnover of c-MYC, suppress HIF1α activity, stabilize p53, and inhibit NF-κB, each of which can have an impact on tumorigenesis.

Circadian clocks orchestrate cellular processes essential for responding to environmental fluctuations, including pathways designated as cancer hallmarks (44) (Figure 2), highlighting how circadian disruption may contribute to malignancy or how transformed cells may hijack circadian clocks to drive malignant growth. This section discusses four circadian-controlled pathways relevant to cancer: cell cycle progression, DNA damage response and repair, hypoxia signaling, and metastasis (Table 1).

### Cell cycle.

Early studies in mice revealed that the timing of cell division in the liver after partial hepatectomy is under circadian control (45). Extensive subsequent work established that even in single cells, circadian and cell cycle oscillators are coupled; the details of this coupling are not well understood and vary with cell type (46–48). Key cell cycle regulators — including cyclins (49) and members of the Cip/Kip family of cyclin-dependent kinase inhibitors (*p21*, *p27*, and *p57*) (50, 51) — exhibit circadian expression across multiple tissues. Public repositories like The Cancer Genome Atlas (52) are a valuable resource for cancer biology; similarly, tools like CircaDB (53) enable researchers to visualize circadian expression patterns for any gene of interest across multiple studies. Such tools provide valuable insights, limited only by the quality of the underlying studies, which may fail to account for sex differences, developmental stages, or interspecies variation, factors that may underlie unresolved links between circadian rhythms and cancer. Regardless of the underlying mechanisms, disruption of circadian influence over cell cycle progression may facilitate unchecked proliferation.

One of the first described links between the circadian clock and cell cycle is WEE1, a kinase that controls the G_2_/M transition by preventing premature mitotic entry. In healthy mice, BMAL1-CLOCK transcriptionally activates *Wee1*, and *Wee1* expression increases in *CRY*-depleted models (45). Ectopic *PER1* expression suppresses *WEE1* independently of p53 in human colon cancer cell lines (54), further suggesting opposing regulation by positive and negative arms of the circadian clock. Consistently, the WEE1-encoding transcript exhibits robust circadian rhythms across many organs in healthy rodents and primates (22, 55–57), consistent with clock-controlled gene dynamics. Notably, circadian rhythmic *Wee1* expression in mouse lungs is disrupted by exposure to chronic jet lag (22). WEE1 protein is transiently enhanced for several days following partial hepatectomy and exhibits daily fluctuations anticorrelated with those observed for BMAL1 protein (58). However, there is disagreement in the literature regarding whether phosphorylation of the WEE1 target CDC2 exhibits daily rhythms correlated with those of WEE1 protein accumulation (45, 58), and it is unclear whether circadian regulation of WEE1 is functionally important for linking circadian and cell cycle oscillations. While *WEE1* is highly expressed in many clinical cases of cancer, it is reportedly absent in colon and non–small cell lung cancers (59), possibly contributing to context-specific effects of circadian disruption on cancer risk.

**Figure 3:**
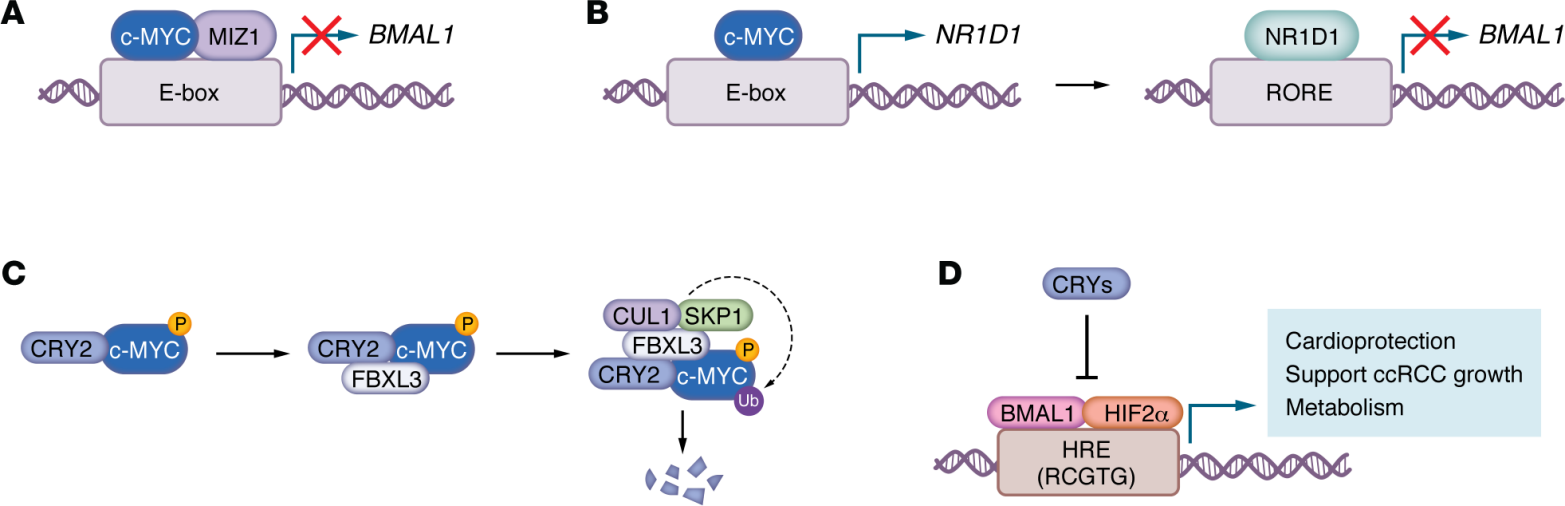
Reciprocal regulation between c-MYC and the circadian clock. (**A**) MYC-MIZ1 directly represses BMAL1 expression. (**B**) MYC indirectly represses BMAL1 by activating *NR1D1* expression. (**C**) CRY2 promotes MYC degradation by recruiting phosphorylated MYC to the SCF^FBXL3^ E3 ubiquitin ligase complex. (**D**) BMAL1-HIF2α regulates physiological and pathological transcription. BMAL1-HIF2α heterodimers promote cardioprotection during myocardial infarction, support ccRCC tumor growth, and drive metabolic gene expression. BMAL1-HIF2α is suppressed by CRY1 and CRY2.

Another key cell cycle regulator is c-MYC, which drives proliferation in many tumors (60). c-MYC and the circadian clock exhibit reciprocal regulation (Figure 3, A–C). Like BMAL1-CLOCK, c-MYC binds E-box motifs, including those regulating *NR1D1* (also known as *Reverba*), a *BMAL1* repressor (61). Thus, c-MYC can interfere with circadian transcription via competitive binding or indirect repression via *NR1D1* activation. Indeed, c-MYC overexpression dampens circadian oscillation of gene expression and of cell physiological processes like glucose uptake through combined upregulation of NR1D1, which represses *BMAL1* transcription (61), and direct repression of *BMAL1* via MYC-MIZ1 complexes (62). While early reports disagreed on whether NR1D1 mediates MYC-induced circadian disruption, MYC consistently increases NR1D1 protein (63, 64). In murine models of colorectal cancer, circadian disruption activates Wnt signaling, a key pathway that promotes *Myc* expression in the intestines (24), which may help explain tissue-specific effects of circadian disruption on tumor growth. Reduced coordination of circadian gene expression is a common feature of many cancers (65). However, some tumors retain robust circadian rhythms (34). It is unknown what determines the degree to which circadian rhythms degrade in tumors, but MYC amplification may promote circadian deregulation. Additional research is needed to understand why tumors exhibit variable disruption of circadian rhythms and whether this has any implications for disease progression or clinical management.

c-MYC typically has a short half-life; its degradation is regulated by multiple pathways (66), including one involving the circadian repressor CRY2. CRY2 recruits phosphorylated c-MYC to SCF-FBXL3, facilitating its turnover in MYC-driven lymphoma (67). WT CRY2 inhibits MYC-driven growth in mouse embryonic fibroblasts, whereas CRY2 mutants identified in patient tumor biopsies that fail to interact with FBXL3 are markedly less effective (33). In vivo, when a human MYC transgene is strongly expressed in mouse lymphoid cells, ubiquitous deletion of *Cry2* leads to more MYC in pretumor spleens and more aggressive lymphoma (67). In contrast, MYC is unaffected in healthy spleens from mice lacking either *Cry1* or *Cry2* in the absence of MYC overexpression, and endogenous MYC is reduced in spleens of *Cry1*^−/−^ *Cry2*^−/−^ mice (68). Additional research is needed to understand these somewhat conflicting findings. The interaction of FBXL3 with the ubiquitination machinery is enhanced by DNA damage (69), so FBXL3- and CRY2-stimulated turnover of MYC may only be relevant in cells subject to MYC-induced replication stress or other sources of DNA damage. Thus, MYC abundance could influence the extent to which it is subject to CRY2-dependent turnover. This noncanonical role for CRY2 in regulating MYC turnover — and the identification of clinically relevant mutations that impair this function — highlights a mechanistic link between circadian disruption and uncontrolled cell proliferation, reinforcing the central role of the circadian clock in cancer physiology. Consistent with this, *CRY2* is expressed at lower levels in many clinical cancer cases compared with matched normal tissue (67), though it remains unclear whether reduced *CRY2* contributes to malignancy or is a consequence of an unknown tumorigenic process. Additional research is needed to investigate whether, and if so how, CRY2 influences tumor formation in a range of contexts (43).

### DNA damage response and repair.

Accumulating evidence links the molecular clock to DNA damage response and repair. Nucleotide excision repair (NER) exhibits rhythmicity (70–72), driven by circadian control of *XPA*, the rate-limiting factor in NER (70–73). Although mammalian cryptochromes (CRYs) have lost their ancestral photolyase activity, mammalian CRY2 preferentially interacts with (6-4) photoproducts (74), potentially supporting noncanonical roles in DNA damage recognition or repair. Indeed, CRY2-deficient cells accumulate DNA damage (32). Further, CLOCK localizes to sites of DNA damage induced by ionizing radiation (75), suggesting that multiple circadian clock proteins may contribute to the DNA damage response. Moreover, DNA damage stabilizes CRY1, destabilizes CRY2, and can reset circadian clocks (31, 32). P53 — a critical mediator of the DNA damage response — binds to the *Per2* promoter, inducing its expression and suppressing BMAL1-CLOCK transcriptional activity (76). Additionally, FBXL3 exhibits enhanced association with CUL1 following etoposide treatment (69), and CRY2 shows increased affinity for FBXL3 in response to doxorubicin (32), highlighting one mechanism of CRY2 destabilization by genotoxic stress. Together, these findings reveal a bidirectional relationship between circadian timing and genomic integrity: circadian dysfunction may impair DNA repair efficiency and increase cancer risk, and DNA damage may perturb circadian rhythms in tumors.

DNA damage triggers apoptosis via p53, preventing the inheritance of corrupted genetic material. As a key tumor suppressor, p53 safeguards genomic stability by halting cell cycle progression in the presence of DNA lesions. PER2 stabilizes p53 by preventing its ubiquitin-mediated degradation (77, 78), while PER1 overexpression enhances p53 nuclear localization and sensitizes cancer cells to DNA damage–induced apoptosis (54). Interestingly, CRY2 mutants identified in tumor biopsies inhibit p53 activity, and in cells with intact P53, they promote unchecked proliferation (33). These findings suggest that the negative arm of the circadian clock promotes genomic stability in part through support of p53 function and that disruption of these components may undermine this tumor-suppressive axis. Activation of p53 by UV light exhibits circadian rhythms, which is dependent on BMAL1 (79). In a murine lung adenocarcinoma model driven by KRAS, circadian disruption by exposure to chronic jet lag (CJL) increases tumor burden (22) more robustly when P53 is intact than when P53 is deleted (21, 22). This may reflect the aggressive nature of P53-deficient tumors masking the effect of CJL or may indicate that the impact of CJL on tumorigenesis involves interfering with one or more tumor-suppressive functions of P53 (21).

When murine hepatocellular carcinoma is induced by ionizing radiation or forms spontaneously, genetic deletion of both *Cry1* and *Cry2* enhances tumorigenesis (19, 20) and renders mice more susceptible to increased tumor formation in response to CJL (19, 80). However, deletion of *Cry1* and *Cry2* reduces tumor formation in *p53*^−*/*−^ mice by resensitizing cells to DNA damage–induced apoptosis (81), suggesting interplay between P53 and CRYs may influence divergent effects of circadian clocks on tumor growth. Together, these studies highlight the complexity of circadian regulation in the DNA damage response and underscore the importance of considering genetic background when evaluating the role of the circadian clock in regulating genomic stability.

### Hypoxia.

Hypoxia arises in solid tumors from rapid cellular proliferation and ensuing competition for oxygen and other nutrients. As tumors grow beyond the capacity of existing vasculature, they develop regions of low oxygen availability — referred to as hypoxic zones — within the tumor microenvironment. Hypoxic stress triggers a cellular response primarily mediated by HIFs. When oxygen is plentiful, prolyl hydroxylase domain enzymes hydroxylate prolines in HIF1α and HIF2α (also known as EPAS1) that promote recognition of HIF1/2α by the von Hippel–Lindau (VHL) tumor suppressor, facilitating ubiquitination and proteasomal degradation of HIFα subunits (82). Under hypoxic conditions — or with *VHL* inactivation, common in ccRCC (83) — this pathway is impaired, leading to stabilization of HIF1α and HIF2α. Stabilized HIFα subunits enter the nucleus, where they dimerize with ARNT (also known as HIF1β) and bind hypoxia response elements (HREs) in the promoters of target genes. These genes regulate biological processes that support tumor adaptation and survival, including angiogenesis (e.g., *VEGFA*), glucose metabolism (e.g., *GLUT1*, *LDHA*), and invasion (e.g., *MMP9, LOX*) (82, 84–86).

HIFs are bHLH-PAS transcription factors and share a close evolutionary and structural relationship with BMAL1 and CLOCK (3, 4). Notably, ARNT and BMAL1 are highly conserved in their bHLH DNA-binding domains and tandem PAS heterodimerization motifs (34, 87). Early reports indicated that BMAL1 could form transcriptionally active heterodimers with HIFα subunits (88), though it was initially believed that BMAL1 was not critical for the hypoxic response. This view was largely based on studies showing BMAL1 was not essential for embryonic angiogenesis (89), a process driven by the HIF-1 complex (ARNT-HIF1α). This long-standing view has shifted in light of accumulating evidence revealing crosstalk between circadian and hypoxia pathways (Figure 3D).

Initial studies on the circadian–hypoxia axis found that in mice, the transcriptional response to hypoxic stimuli in liver, kidney, skeletal muscles, and lung is heavily influenced by the time of day at which the stimulus is delivered (90–93). The effect of exposure to hypoxia at high altitude on the human blood transcriptome also depends on the time of day, suggesting that this phenomenon is conserved across species (94). PER- and CRY-deficient murine fibroblasts and skeletal muscles showed greater induction of hypoxia-targeted gene expression compared with WT controls upon exposure to HIF-activating stimuli (90, 95), including pro-apoptotic genes *Bnip3* and *Noxa1*, suggesting a protective role for circadian clock repressors during hypoxia-induced apoptosis (90). These findings indicate that circadian regulation of hypoxic gene expression is time of day dependent, tissue specific, and influenced by the molecular clock.

Circadian expression of *Hif1a* (90, 96) and *Hif2a* (97) could contribute to circadian gating of the transcriptional response to hypoxia. However, sequence and structural homology between circadian and hypoxia transcription factors led to the discovery that BMAL1 dimerizes with HIFα subunits. BMAL1-HIFα heterodimers enable transcriptional activation of genes containing HREs (34, 95) — motifs that closely resemble the E-box sequences bound by BMAL1-CLOCK (Figure 3D). BMAL1 colocalizes with HIF1α (90) and HIF2α (34) in cells at a number of endogenous chromatin sites, indicating extensive crosstalk between circadian- and hypoxia-responsive transcriptional networks. Notably, BMAL1 contributes to HIF-driven gene expression in disease contexts, including diet-induced obesity (98) and kidney cancer (34). More recently, the BMAL1-HIF2α heterodimer has been implicated in cardioprotection during myocardial infarction (35) and in promoting ccRCC growth (34). BMAL2 apparently contributes to hypoxia-induced gene expression and metabolic reprogramming in pancreatic cancer, potentially through interactions with HIF1α (87), further highlighting the convergence of these regulatory pathways in pathophysiological processes. Notably, BMAL2 expression is higher in tumors compared with adjacent normal samples across many tumor types (99). It is unclear whether or how BMAL2 contributes to tumorigenesis.

Circadian clock repressors can inhibit HIF-mediated transcription. Cryptochromes suppress HIF1α target genes (95, 100) and repress the transactivation of BMAL1-HIF1α heterodimers (95). Loss of CRYs enhances fatty acid oxidation (28) and glycolysis (95) in mouse myotubes, likely due to enhanced activity of nuclear hormone receptors and HIF, respectively. CRY1-deficient mouse embryonic fibroblasts exhibited enhanced proliferation and migration in one study that attributed these effects to increased HIF-1 activity (100). However, there is disagreement in the literature regarding the impact of CRY1-depletion on proliferation in fibroblasts (67), and other studies found that CRY1 promotes growth in prostate cancer cells (31). Regardless, accumulating evidence supports the idea that CRYs limit HIF-driven transcription, including of tumor-promoting pathways. These findings suggest that CRYs may have a protective role in hypoxia-driven tumors, potentially limiting tumor progression under low oxygen or other conditions of HIF stabilization.

HIFs play a critical role in normal physiology, particularly in kidney and brain, which are finely attuned to oxygen fluctuations. In tumors like ccRCC and GBM, HIFs function not merely as stress-responsive factors but as central drivers of tumor identity, progression, and therapeutic resistance. These malignancies uniquely depend on genetic (83, 86, 101–103) and/or oncogenic signaling-driven (104) mechanisms that stabilize HIFs. Notably, both ccRCC and GBM exhibit preferential reliance on HIF2α (104–106) over HIF1α, distinguishing them from other tumor types where HIFs are either inactive, stabilized solely by microenvironmental hypoxia, or more dependent on HIF1α activity. This may help explain why tumors like ccRCC (34) and GBM (39) exhibit impaired growth upon BMAL1 loss, whereas other cancers do not (21, 24). This hypothesis warrants further investigation to elucidate the mechanistic interplay between the molecular clock and tumorigenesis and may uncover novel therapeutic opportunities that leverage the circadian–hypoxia axis in specific cancer contexts.

Conversely, HIFα stabilization can modulate circadian rhythms. In rodents, physiological oxygen levels oscillate daily; similar fluctuations can reset circadian clocks in cultured cells in a HIF1α-dependent manner (107). This suggests that oxygen-driven HIF1α activation may serve as a Zeitgeber — an environmental timing cue — for the circadian system in some mammals. However, chronic HIF1α stabilization disrupts circadian rhythms across multiple models, including murine skeletal muscle (93), U2OS human osteosarcoma cells (90), and NIH3T3 mouse embryonic fibroblasts (100). In contrast, ccRCC cells, in which HIF2α is stabilized due to VHL inactivation, display robust circadian rhythms that are dampened upon reintroduction of WT VHL (34). This observation suggests that, unlike HIF1α, HIF2α may promote rather than disrupt circadian rhythmicity. Supporting this, a naturally occurring variant of HIF2α in the high-altitude–adapted plateau pika dampens circadian rhythms, whereas in rodents that do not natively inhabit high-altitude environments, HIF2α does not exhibit this suppressive effect (108). These findings raise the possibility that HIF2α may support circadian oscillations, in contrast to the disruptive influence of sustained HIF1α activity. Thus, HIF1α and HIF2α may exert distinct, and potentially opposing, effects on circadian rhythm regulation. Alternatively, differences in the mode of HIF stabilization — whether driven by drugs, VHL inactivation, exercise, or hypoxia — may differentially impact circadian clock function. Further mechanistic studies are needed to clarify how specific HIF isoforms and stabilization contexts modulate circadian rhythms and how these effects intersect with tumor progression and treatment responses.

### Metastasis.

Several recent findings suggest the timing of metastasis may be governed by daily rhythms. In human and mouse breast cancer models, spontaneous shedding of circulating tumor cells peaked during the rest phase of the circadian cycle (i.e., sleep), and tumor cells released during this phase exhibited enhanced expression of mitotic genes, suggesting that not only are more tumor cells circulating during sleep but the shed cells also have enhanced metastatic proficiency (109). In luminal A breast tumors, robust circadian rhythms correlated with increased epithelial-to-mesenchymal gene cycling, higher metastatic potential, and poorer patient survival (110).

While these studies highlight compelling rhythms in metastatic behavior, the specific roles of sleep versus underlying circadian phase remain to be distinguished. Sleep per se could mechanistically contribute via hormone fluctuations, immune suppression, or altered vascular permeability during rest. Conversely, circadian regulation independent of sleep might govern transcriptional programs that prime cells for intravasation or dissemination. Disentangling these factors will require controlled designs that separately interrogate sleep and endogenous clock phase, such as repeated sampling anchored to circadian markers (e.g., melatonin onset) and concurrent sleep–wake monitoring.

## Chronotherapy

**Figure 4:**
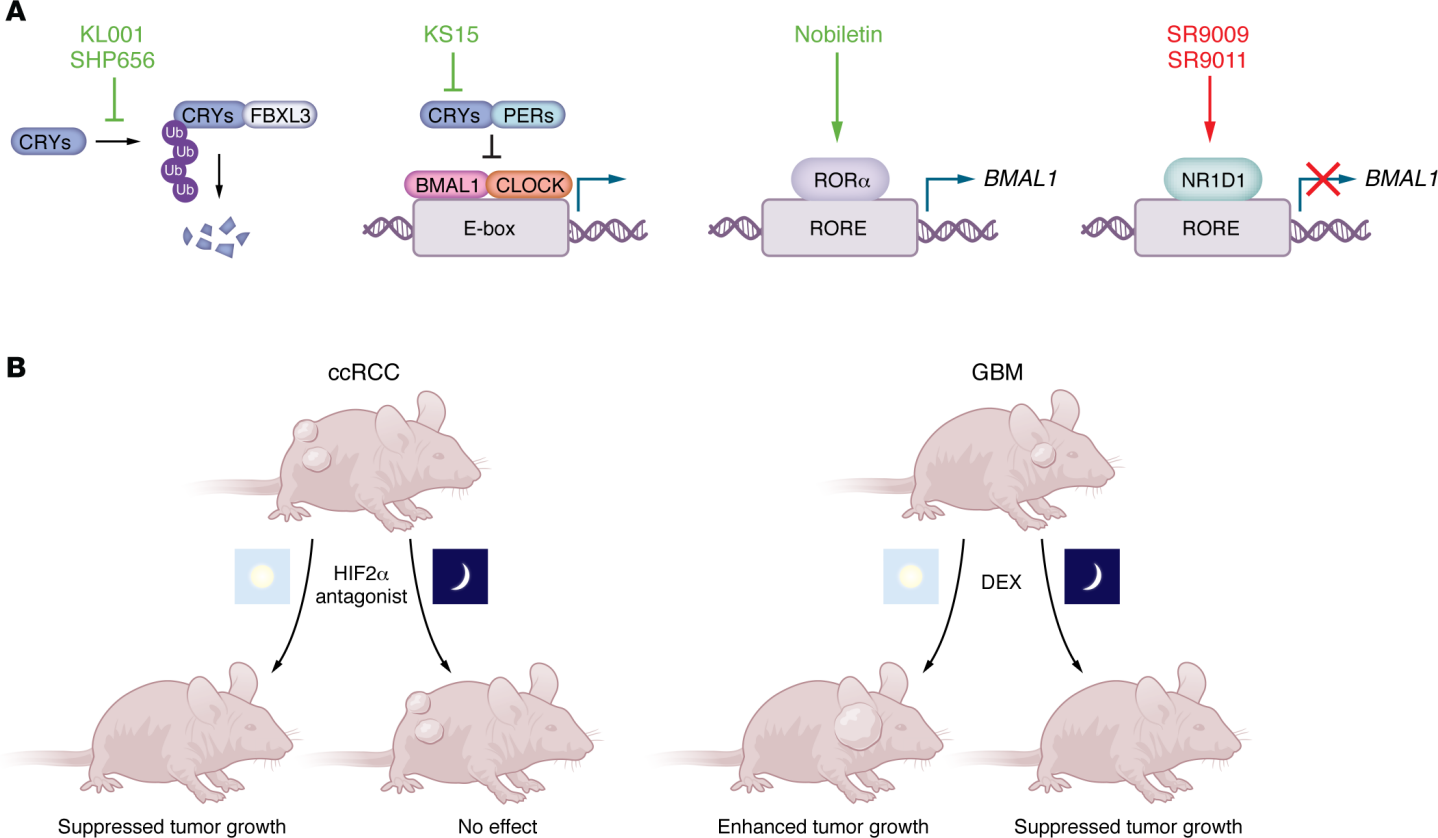
Chronotherapy strategies in cancer. (**A**) Representative small molecules targeting circadian clock proteins. (**B**) Time of day–dependent efficacy of a HIF2α antagonist (PT2399) in a ccRCC model and of DEX treatment in a model of GBM. PT2399 treatment of mice with ccRCC xenograft tumors at the beginning of the light phase suppresses tumor growth, whereas dosing at the onset of the dark phase is ineffective. Conversely, DEX treatment of mice with GBM tumors during the light phase enhances tumor growth, while DEX treatment during the dark phase reduces GBM xenograft growth.

Chronotherapy leverages circadian rhythms to optimize therapeutic efficacy and minimize adverse effects. This approach recognizes that the timing of drug administration — relative to an individual’s circadian phase — can significantly affect treatment outcomes. Chronotherapy can be broadly divided into three interconnected strategies: (a) training clocks, which involves strengthening endogenous circadian rhythms through lifestyle interventions such as regulated sleep, diet, and physical activity; (b) drugging clocks, which focuses on using small molecules to target components of the molecular clock; and (c) clocking medicine, which determines the optimal time of day for drug delivery to maximize efficacy and reduce toxicity (111). Here, we focus on the latter two strategies: drugging clocks and clocking medicine (Figure 4).

### Drugging clocks: small molecules targeting circadian clock components.

Cancer therapy faces persistent challenges, including tumor heterogeneity, genomic instability, and the emergence of resistance. Targeting the circadian clock offers a novel strategy to overcome some of these obstacles by exploiting tumor-specific vulnerabilities in temporal regulation of molecular clock proteins and clock-controlled genes. While work in this area spans multiple tumor types, GBM has emerged as a key model, with promising applications in other malignancies, including ccRCC.

GBM is largely resistant to conventional therapies. BMAL1 and CLOCK drive cell cycle progression, inhibit apoptosis, and maintain metabolic homeostasis in GBM stem cells (GSCs) derived from clinical samples (39). Targeting these factors may thus present a viable therapeutic strategy. REV-ERBα agonists SR9009 and SR9011 reduce *BMAL1* expression and suppress GSC proliferation (39) and GBM xenograft growth in vivo (112). SR9009 also reduces *BMAL1* expression and induces autophagy in small cell lung cancer cell lines but not in normal human bronchial epithelial cells (113). However, SR9009 treatment reduced viability and altered gene expression in hepatocytes and embryonic stem cells regardless of the presence or absence of REV-ERBα and REV-ERBβ, suggesting that these responses to SR9009 may not be mediated by REV-ERBs (114).

Another promising strategy involves stabilizing CRY proteins by blocking their interaction with FBXL3, preventing their proteasomal degradation (115–117). The CRY-stabilizing compounds KL001 and SHP656 have antiproliferative effects in GSCs in cell culture (39). SHP656 also enhances the efficacy of bevacizumab, an anti-VEGFA antibody, in colorectal cancer xenografts (118), and improves response to anti–PD-1 immunotherapy while independently slowing tumor growth in preclinical models (119). CRY-stabilizing compounds have been deemed safe in phase I clinical trials, highlighting their clinical potential (120). The CRY inhibitor KS15, which disrupts CRY–BMAL1 interactions (121), suppresses growth and enhances chemosensitivity in breast cancer cell lines (122), demonstrating that both CRY inhibition and stabilization may be effective depending on tumor context.

Nobiletin (NOB), a natural compound derived from *Citrus reticulata* and *aurantium*, activates RORα and enhances *BMAL1* expression (123). NOB reduces cell proliferation and induces cell cycle arrest in several breast cancer cell lines, while NOB treatment of OVCAR3 ovarian cancer cells enhanced apoptosis in vitro and reduced angiogenesis and tumor growth in OVCAR3-based xenografts in athymic mice (124–126). In gastric cancer cells, NOB reduces proliferation, arrests the cell cycle, and induces apoptosis (127). In two cell lines derived from different types of kidney tumors (ACHN and Caki-2), NOB disrupts oncogenic signaling, suppresses proliferation, and induces cell cycle arrest and apoptosis (128). Furthermore, NOB treatment in vivo robustly reduced the growth of ACHN-based xenograft tumors in nude mice (128). Taken together, these studies suggest that NOB may slow growth of several tumor types. Notably, none of these studies investigated whether BMAL1 is required for the effects of NOB. Interestingly, NOB-driven suppression of ACHN and Caki-2 cell growth appears to contrast with reduced growth of ccRCC cell lines in which BMAL1 is depleted (34). These differences may be explained by distinct genetic contexts: the RCC cell lines used in the NOB study either express WT VHL (ACHN) or lack HIF2α (Caki-2) (128), whereas the BMAL1 dependency study focused on VHL-deficient, HIF2α-driven ccRCC models (34). These findings highlight the context-dependent effects of circadian modulation and support the broader therapeutic potential of circadian clock–enhancing compounds like NOB, particularly when tumor-specific circadian wiring and oncogenic dependencies are considered.

Importantly, many of the studies discussed compare clock-targeting compounds only to vehicle controls rather than standard-of-care chemotherapies, and few evaluate their potential in combination therapy settings. These gaps highlight the need for future studies to determine how these compounds may best complement existing treatment regimens.

### Clocking medicine: optimal treatment times for targeted therapies.

Comprehensive reviews of clinical trials testing time-of-day effects on outcomes for chemotherapy, radiation therapy, immune checkpoint inhibitors (ICIs), and first-line targeted chemotherapeutics are available elsewhere (129–132). Notably, some studies report conflicting results, for example, with temozolomide in GBM (133–135), likely reflecting differences in study design, anchoring to clock time versus chronotype, molecular subtypes, and selected endpoints. With growing recognition that treatment time can influence outcomes, major challenges remain to enable clinical implementation of insights from circadian biology to improve cancer treatment. Here, we discuss recent preclinical research findings that reveal mechanistic explanations for divergent responses to targeted treatments based on the time of day of drug delivery.

Glucocorticoids are commonly used as adjunctive therapy in patients with GBM to alleviate symptoms of cerebral edema (136, 137). Dexamethasone (DEX) is preferred due to its potent anti-inflammatory effects and ability to cross the blood–brain barrier (137). Interestingly, some studies reported that DEX suppresses tumor growth, while others showed that it can enhance glioma proliferation (138). The variability may be partially explained by interactions between glucocorticoid receptor (GR) signaling and the circadian clock. GR is regulated by core circadian clock components (139), suggesting that the timing of DEX administration could influence therapeutic outcomes. Supporting this, in GBM xenograft models, DEX administration in the morning (when *Per2* is low) enhances tumor growth, whereas evening administration (when *Per2* is high) suppresses it (140). Similarly, in breast cancer, where DEX is used to mitigate chemotherapy-induced side effects, DEX has antitumor activity in some contexts, but other studies find that it promotes breast cancer cell proliferation and metastasis (141). These divergent effects, as in GBM, may be influenced by circadian timing. Together, these findings highlight the importance of considering circadian phase in the administration of glucocorticoids, particularly when used as adjunctive therapy in cancer. Optimizing the timing of DEX delivery could enhance therapeutic efficacy while minimizing unintended protumorigenic effects.

Recent findings indicate that dosing time could also influence outcomes for belzutifan, a relatively new treatment for ccRCC. ccRCC is characterized by loss of VHL (142), which targets HIF1α and HIF2α for degradation. VHL suppresses ccRCC growth through inhibition of HIF2α activity (105, 106), underscoring the oncogenic function of HIF2α in ccRCC. Given its central role in tumor progression, extensive efforts have focused on developing a compound that inhibits HIF2α-ARNT dimerization. This led to the 2021 FDA approval of belzutifan (previously called PT2977) for VHL-null ccRCC (143). However, resistance remains a major challenge, as nearly one-third of ccRCC patient-derived xenografts (PDXs) fail to respond to HIF2α antagonists such as PT2399, which is closely related to belzutifan (105, 144). BMAL1 is highly expressed in ccRCC clinical samples, and BMAL1-HIF2α heterodimers support tumor growth in xenograft models (34). PDXs that are sensitive to growth inhibition by PT2399 express higher levels of *BMAL1* relative to resistant tumor grafts (144), and cell line–derived xenograft models are sensitive to suppression by PT2399 when the drug is administered at the beginning of the light phase — when BMAL1 expression is high — and resistant when mice are dosed at the beginning of the dark phase (34). This suggests that outcomes for patients treated with belzutifan may be improved by treating them when *BMAL1* expression is high in their tumors. If NOB treatment enhances *BMAL1* expression in ccRCC, HIF2α antagonist and NOB combination therapy may result in improved outcomes. Further research is needed to explore these possibilities.

Harvesting the immune system’s killing capacity is a powerful therapeutic strategy across several cancers, often yielding persistent responses (145). Circadian rhythms regulate immune functions, driving oscillations in leukocyte levels, tissue recruitment, and inflammatory activity — factors that shape both acute and chronic disease outcomes and highlight the importance of intervention timing (146). Endogenous leukocyte rhythms, oscillations in tumor immune infiltration, and circadian clocks within endothelial cells in the tumor microenvironment influence tumor growth and responses to immunotherapies including ICIs and CAR T cell therapy in preclinical models (147). In melanoma, lung, and colorectal cancer models, anti–PD-1 and anti–PD-L1 therapies are more effective when administered to mice in the early active phase (38, 147). Consistent with preclinical research, patients with advanced gastric (148), kidney (149), or melanoma (150) cancers have improved outcomes with morning dosing of ICI compared with patients treated later in the day. Inconsistent results have been reported regarding optimal ICI timing in non–small cell lung cancer, with one retrospective clinical study showing longer survival with late-day treatment (151), and another reporting better outcomes with early administration in patients (152), suggesting factors such as chronotype, tumor-specific circadian profiles, or patient lifestyle may influence therapeutic window. Additional investigation is needed to replicate these findings in additional centers and to understand how responses to ICI are influenced by circadian time in patients with diverse tumor types to support the integration of chronotherapy into future cancer treatment strategies.

## Conclusions and future directions

Circadian rhythms regulate key physiological processes that influence cancer development and progression, including cell cycle progression, DNA damage repair, metabolism, immune surveillance, hypoxic adaptation, and metastasis. Disruption of circadian rhythms can significantly alter tumor growth and therapeutic responses. However, the impact is highly context dependent, highlighting the need for deeper mechanistic studies investigating the role of the circadian clock across a range of cancer types.

Directly targeting circadian components is an emerging and promising strategy to suppress tumor growth. Small molecules like REV-ERB agonists, CRY stabilizers, and NOB demonstrate antitumor effects. However, because the clock supports normal tissue homeostasis, systemic inhibition with these agents may pose toxicity risks. Targeting molecular clock proteins selectively hijacked by tumors (e.g., BMAL1-HIF2α in ccRCC or CRY2-mediated MYC degradation in lymphoma) may offer improved safety and efficacy.

Clinical translation of circadian medicine faces barriers, including logistical challenges of timed therapy, variability in individual rhythms, and the need for robust biomarkers like blood-based chronotyping. Prospective trials that incorporate patient chronotype and tumor-specific circadian profiles will be key to advancing the field. Such efforts could unlock safer, more effective use of cytotoxic, targeted, and immune therapies in oncology.

The circadian clock is a powerful yet context-dependent modulator of cancer biology. Its complex roles demand a cancer-specific approach to clinical translation. Future efforts should focus on integrating circadian profiling into diagnostics, refining selective chronotherapeutics, and designing clinical trials that consider time-of-day effects. Such strategies are key to unlocking the full potential of circadian medicine in the era of precision oncology.

## Funding support

This work is the result of NIH funding, in whole or in part, and is subject to the NIH Public Access Policy. Through acceptance of this federal funding, the NIH has been given a right to make the work publicly available in PubMed Central.

- National Cancer Institute grants CA211187 and CA271500 (to KAL).
- National Cancer Institute grants R01CA244519 and R01CA259370 (to SM).

## Figures and Tables

**Table 1:**
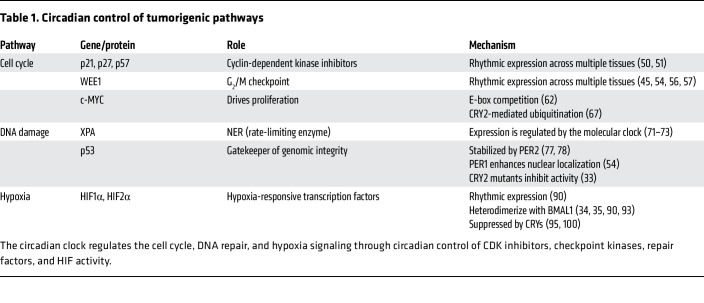
Circadian control of tumorigenic pathways

## Version 1 02/02/2026

Electronic publication
